# Decoding France’s food environment combining data bases to characterize the food environment in France

**DOI:** 10.1016/j.dib.2025.111850

**Published:** 2025-07-11

**Authors:** Quentin Creurer, Simon Vonthron, Mohamed Hilal, Helene Charreire, Claude Napoleone, Manon Pradere, Esther Sanz-Sanz

**Affiliations:** a INRAE, UR Ecodéveloppement, Avignon, France; b INNOVATION, Univ Montpellier, CIRAD, INRAE, Institut Agro, Montpellier, France; c UMR CESAER, INRAE, Institut Agro, Dijon, France; d MoISA, Univ Montpellier, CIRAD, CIHEAM-IAMM, INRAE, Institut Agro, IRD, Montpellier, France

**Keywords:** Food environment, French Food outlets Data Base, Spatial Data Base

## Abstract

The dataset presented here draws on several French institutional database sources. The aim is to develop a nationwide database of food outlet locations that can be automatically enriched and updated with a high level of reliability. To this end, we created a flexible and dynamic Python script that can be widely utilized by a maximum of users. Data can be updated either on a daily basis to create a series of files within a limited timeframe, or on a monthly basis to reflect updates to the SIRENE 3.11 database of the national statistical institute INSEE. Starting from SIRENE 3.11, we enriched the data using the *Alim’ Confiance* (Ministry of Agriculture) and the *Les Professionnels Engagés en Bio* (organic farmers) databases. We were also able to enhance the accuracy of food environment description by identifying food outlets currently closed, exploiting the BODACC (official trade bulletin) database with its almost daily updating. These data provide valuable support for studies on the food environment at both macro and micro levels. The database’s wide range of scales makes it possible to map food sales and distribution outlets, a prerequisite to any investigation of food environments in a given territory.

Specifications Table

**Table:** 

Subject	Computer Sciences
Specific subject area	Food retail density, diversity, and access
Type of data	CSV file, Jupyter notebook file, Python file, Database
Data collection	Online acquisition through governmental databases and APIs
Data source location	INSEE; France
Data accessibility	Repository name: French Food Outlets Script Data identification number: 10.5281/zenodo.14882280 Direct URL to data: Zenodo. https://doi.org/10.5281/zenodo.15035782

## 1 Value of the Data

• Data from SIRENE 3.11 are upgraded through the Agence Bio and Alim’ Confiance databases providing a new set of variables regarding the quality of the distributors (organic food…).
• Data are regularly updated, using the BODACC database to identify food outlets that have been closed giving a closer look at the real situation on the ground.
• Database is stored on a github platform accessible to anyone who is willing to improve the coding aspect of the research. We have been building a robust database and dataset over time aiming of reaching as many people as possible interested in food environment issues.
• Data are dynamic giving that there is room for improvement through the addition of Python scripts adding new databases or update from the databases used.
• All the datas gathered are under the Open Licence which give us and others the ability of manipulated the datas and redistribute it to the greatest number of people.

## 2 Background

Numerous studies have highlighted the critical role that the food environment plays in shaping consumer choices and individual food behaviors [[1], [2], [3]]. Food environment can be defined as “the settings with all the different types of food made available and accessible to people as they go about their daily lives”.

In France, while limited, food environment research is increasingly emerging [[4], [5], [6], [7]]. However, exhaustive large-scale field surveys (e.g., regional or national) are impractical due to the significant cost and time involved. Thus, French food environment studies are mainly based on secondary.

To fill this gap, we built a robust spatial database that provides information on food outlets. First, we improved it by cleaning the data from SIRENE 3.11. For example, we reassigned variables to classes: the variable SIRET can, for instance, appear as a number whereas it is actually a string.

Second, we added information on organic food retail outlets from existing open French databases such as Alim’ Confiance and Les Professionnels Engagés en Bio (Professionals Committed to Organic Farming). Lastly, we updated the data by identifying closed food outlets based on the BODACC database (The Official Bulletin of Civil and Commercial Announcements), which is more accurate than SIRENE 3.11 regarding the parameter *open or closed food outlets*.

## 3 Data Description

The initial data come from various French administrative services and are available on the open Datagouv platform. **T**he SIRENE 3.11 database is produced by the French National Institute of Statistics and Economic Studies (INSEE), while the Alim’ Confiance Data Base is generated by the French ministry of Agriculture and Food. Les Professionnels Engagés en Bio data on professionals involved in organic farming is managed by Agence Bio, the French agency for the development and promotion of organic farming established in 2001. Lastly, the BODACC database is produced by the Prime Minister’s Office through the Directorate for Legal and Administrative Information (DILA).

The SIRENE 3.11 database encompasses all facilities surveyed in France, regardless of their activities or legal structure. This includes both public and private facilities, as well as foreign companies operating within France. SIRENE 3.11 provides an overview through its nearly 40 million entries.

The establishment’s stock file is updated on the 1^st^ of every month. Version 3.11 incorporates addresses and geolocation data (Lambert coordinates) derived from the National Address Database (BAN), a comprehensive database of geo-referenced addresses in France. Each facility is identified by a unique identification code: the SIRET number. The various types of activities reported in the database are identified through an APE code indicating main activity.

**Fig. 1:**
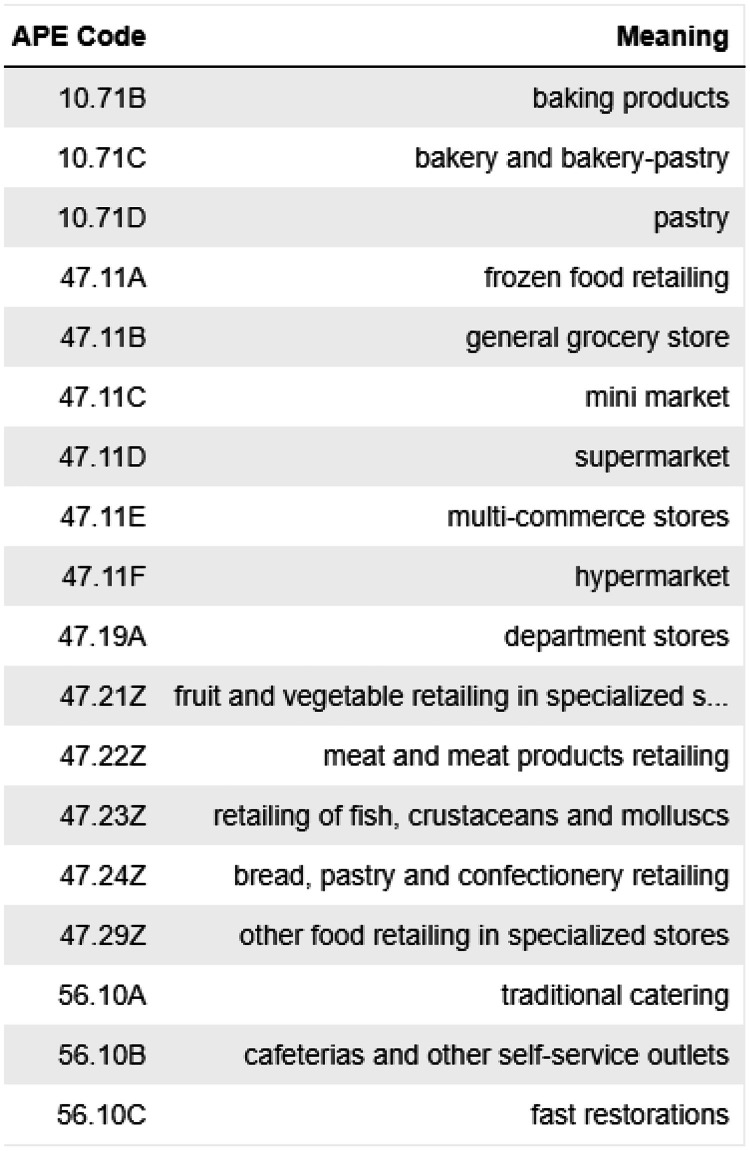
APE code for food outlets in SIRENE 3.11

After filtering based on APE codes (Fig. 1) for types of food outlets (e.g., supermarket, grocery store, restaurant), we obtain a database containing around 500,000 entities, i.e., 1.25 % of the total SIRENE 3.11 database.

It is important to note the distinction between SIRET and SIREN numbers. The SIRET number (Système d’Identification du Répertoire des Etablissements) is a 14-digit code composed of the 9-digit SIREN number (Système d’idenfication du repertoire des entreprises) and the 5-digit NIC (Numéro interne du classement Insee). Essentially, the SIRET number identifies a specific establishment, while the SIREN number refers to the company itself.

The Alim’ Confiance database is compiled from hygiene inspections conducted to enhance consumer confidence in the food sector. All food outlets, including stores, restaurants, and slaughterhouses, are potentially inspected. They are identified by their SIRET number. This dataset provides geolocation information, as inspections are carried out on-site. This yields full information on business names for some 12,000 food outlets in the Alim’ Confiance database.

Agence Bio provides a database of economic operators involved in organic farming and registered with Agence Bio. In this database we can find informations such as SIRET numbers that we can link to business names and dates of establishment, for instance.

The BODACC enables a more accurate assessment of which food outlets are truly open. It is updated five times a week with official notices issued by commercial court clerks, civil courts with commercial jurisdiction, or judicial representatives. This helps to filter out food outlets that are actually closed but still included in the SIRENE 3.11 database.

This dataset is of particular interest for future research into food retailers specialising in organic products, as there is a process underway in the Agence Bio database to complete it and improve the quality of the variables. Our work lays the foundations and serves as a relay for other research, both in terms of the food landscape and the technical work of creating a database from the various French government bodies, in this case through the API developed.

It is also interesting to note that the use of the BODACC opens the door to future research, not only on the delisting of food businesses, but also on the types of change in food businesses, with the aim of observing both quantitative and qualitative change. A takeover of a traditional restaurant may result in a change of APE code to become a fast-food outlet, and it would be interesting to study this potential phenomenon (Table 1).

**Table 1:** Sources, producers and file names of databases.

Source	Producer	File Name
Base Sirene des entreprises et de leurs établissements	INSEE	StockEtablissement_utf8.zip
Alim’ Confiance	Ministère de l’Agriculture et de l’Alimentation	export_alimconfiance.csv
Professionnels engagés en BIO	Agence Bio	operateursbio.csv
API BODACC	DILA	annonces-commerciales.csv

## 4 Experimental Design, Materials and Methods

Our aims are to I) enhance geolocation of food outlets, II) update information on food outlets by identifying and deleting closed food outlets), and III) add if food outlets has organic food products to the SIRENE database, using other existing databases.

The first methodological challenge was to merge and harmonize all the existing relevant databases, enabling users to dynamically update as needed. First, we identified the SIRET number as the primary key allowing the Alim’ Confiance and Agence Bio databases to be linked to the SIRENE 3.11 database. However, food outlets listed in SIRENE but marked as closed in the BODACC database could not be identified, due to the fact that the SIRET number is not included in BODACC. To address this, we used the SIREN number to merge BODACC and SIRENE 3.11.

For instance, the international discount retailer Lidl is represented by SIREN code **343262622**, whereas there are multiple SIRET codes for the individual Lidl supermarkets, one for each establishment. For instance, SIRET **343262622***00586* corresponds to a Lidl supermarket in Strasbourg, while **3432626222***6771* is a Lidl supermarket in the city of Gap.

### 4.1 Structure of the harmonized food outlet database

Our newly generated database is rooted in the robust and reliable SIRENE 3.11 database, which is updated monthly. Its extensive data volume provides a solid foundation for our analysis. To build the database, we automated a cleaning and enrichment process using a Python script. We were also aiming for a flexible approach that would allow users to update the database, potentially on a daily basis, although this may not be necessary considering the BODACC database’s update frequency of 5 times a week.

We used the Polars library, which accelerates the automation of import, filtering, and cleaning tasks. A pre-filtering mechanism reduces the amount of data processed, further optimizing performance by processing around 500,000 entities instead of 40 million. Leveraging French government APIs and the efficiency of Polars, an 8 GB file can be filtered to include only food outlets in under 2 minutes. The entire script generates a complete CSV file of food outlets in less than 10 minutes. At the same time, Alim’ Confiance and Agence Bio files are stored and annotated with the current date or month, enabling temporal and dynamic database management.

**Fig. 2:**
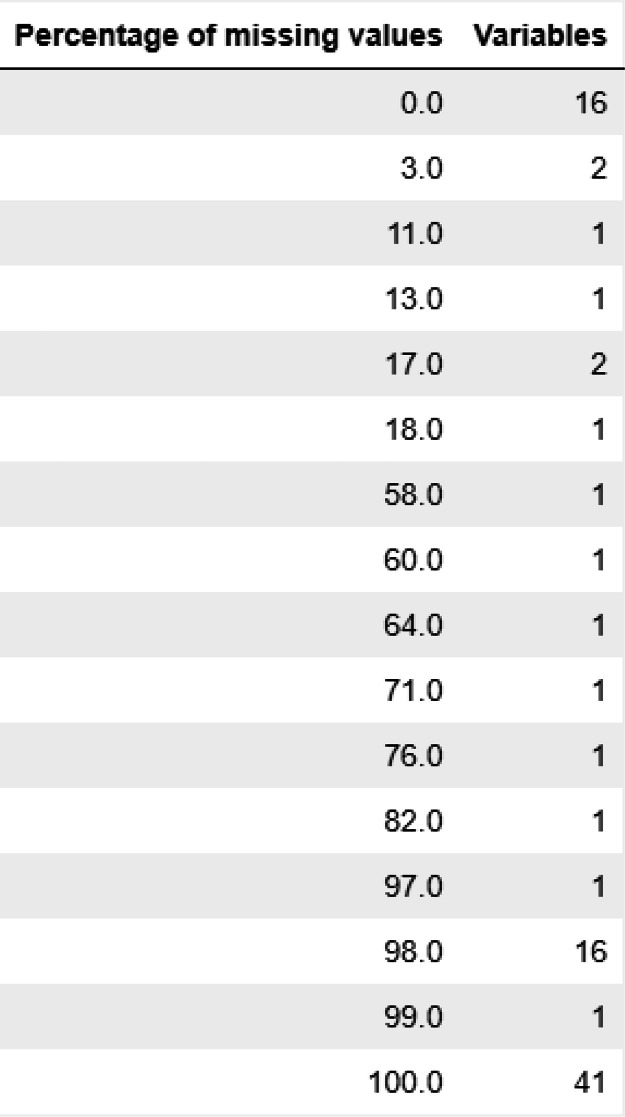
Percentages of missing values per numbers of variables

However, the CSV file still contains a large number of errors on composition variables and a large number of missing values. We observe that 59 variables have a percentage of missing values greater than 90 %, including 41 variables with no data (Fig. 2). It should be noted that some of the variables with fields not filled in are either not relevant to the food environment or are variables for which the information does not exist (e.g., secondary or tertiary address). Two variables from the BODACC stand out as not relevant to food environment research: “Jugement” (“*A decision handed down by a court (a court of first instance) when it rules in a collegial manner (i.e., by three magistrates), or as a single judge (a single magistrate). When a decision is handed down by a court of appeal or by the Cour de cassation, it is referred to as a judgment*.) and “Agrement”.

Moreover, 17 % of values are missing for the geographic coordinates, our major variable. This is generally due to the transition from the SIRENE 3.9 database to 3.11, which will stabilize at version 4 with fully usable coordinates. INSEE and IGN (National Institute of Geographic and Forest Information) data can be expected to gradually correct these issues with the geo-coordinate variables, eventually leading to version 4 in March 2026.

Nonetheless, fields are fully filled on 16 variables (Fig. 2), primarily for the variables SIRET and its components SIREN and NIC, both extremely useful in merging these databases. The 16 include key variables such as *activitePrincipaleEtablissement* and *etatAdministratifEtablissement*, which enabled us to distinguish SIRENE 3.11 food outlets still open, as well as the variable we created, *etat_etablissement_bodacc*. The other variables in this list are, for the time being, of less interest as a tool for the construction of the database.

16 variables with fields fully filled:

- ActivitePrincipaleEtablissement
- CaractereEmployeurEtablissement
- CodeCommuneEtablissement
- DateCreationEtablissement_x
- DateDebut
- DateDernierTraitementEtablissement
- EtablissementSiege
- EtatAdministratifEtablissement
- Etat_etablissement_bodacc
- LibelleCommuneEtablissement
- Nic
- NombrePeriodesEtablissement
- Nomenclature Activite Principale Etablissement
- Siren
- Siret
- StatutDiffusionEtablissement

### 4.2 Descriptive statistics

All statistics in this section are derived from the database generated on December 18, 2024. This database comprises 495,944 food outlets and 89 variables. While some may not be relevant, they contribute to a comprehensive understanding of the database structure involving new implementations from INSEE throughout the process toward SIRENE 4.

To further develop this database, we are particularly interested in two variables: the date of the business’s creation and the date of the last INSEE update. Additionally, Lambert coordinate variables are crucial for spatially locating food stores.

To illustrate the potential applications of this database, we have created data visualizations representing fundamental aspects of the French food environment, such as the distribution of various food retail and restaurant categories based on the APE code (i.e., main declared activity of the business).

**Fig. 3:**
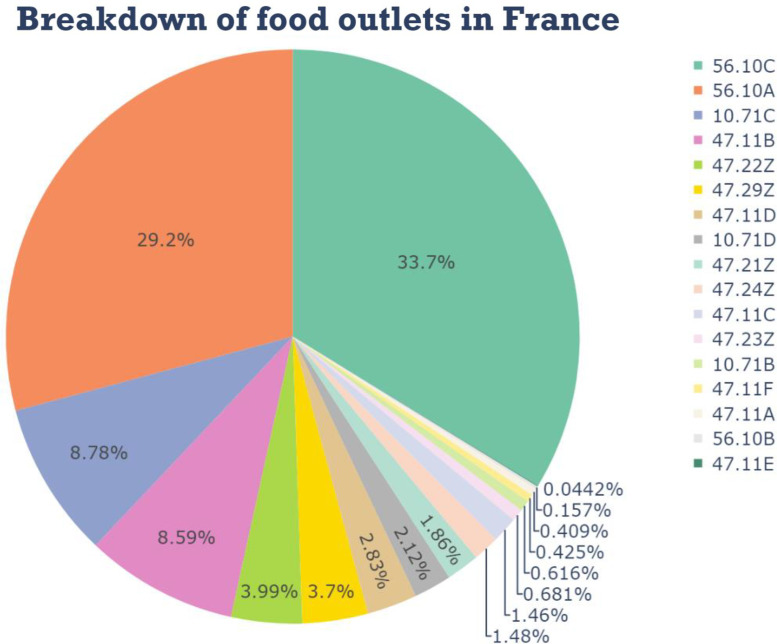
Breakdown of France’s food outlets by APE code

Fig. 3 describes the distribution of food outlets in November 2024 from our harmonized database. Notably, traditional restaurants (APE code: 56.10A) and fast-food restaurants (APE code: 56.10C) collectively account for over two-thirds of the market.

The integration of data from Alim’ Confiance, Agence Bio, and BODACC significantly enhances the accuracy of SIRENE 3.11 regarding the distribution of food outlets.

As of December 18, 2024, our database identified 495,240 food outlets. However, by leveraging the BODACC database, we were able to identify 3,570 (approximately 0.75 %) of these establishments as closed, despite their presence in the SIRENE 3.11 database.

To delve deeper into these closures, we extracted data for the 10 cities with the highest number of food outlets in France. This sub-dataset includes city name, total number of food outlets, number of closed food outlets (BODACC) and percentage of closed food outlets.

**Fig. 4:**
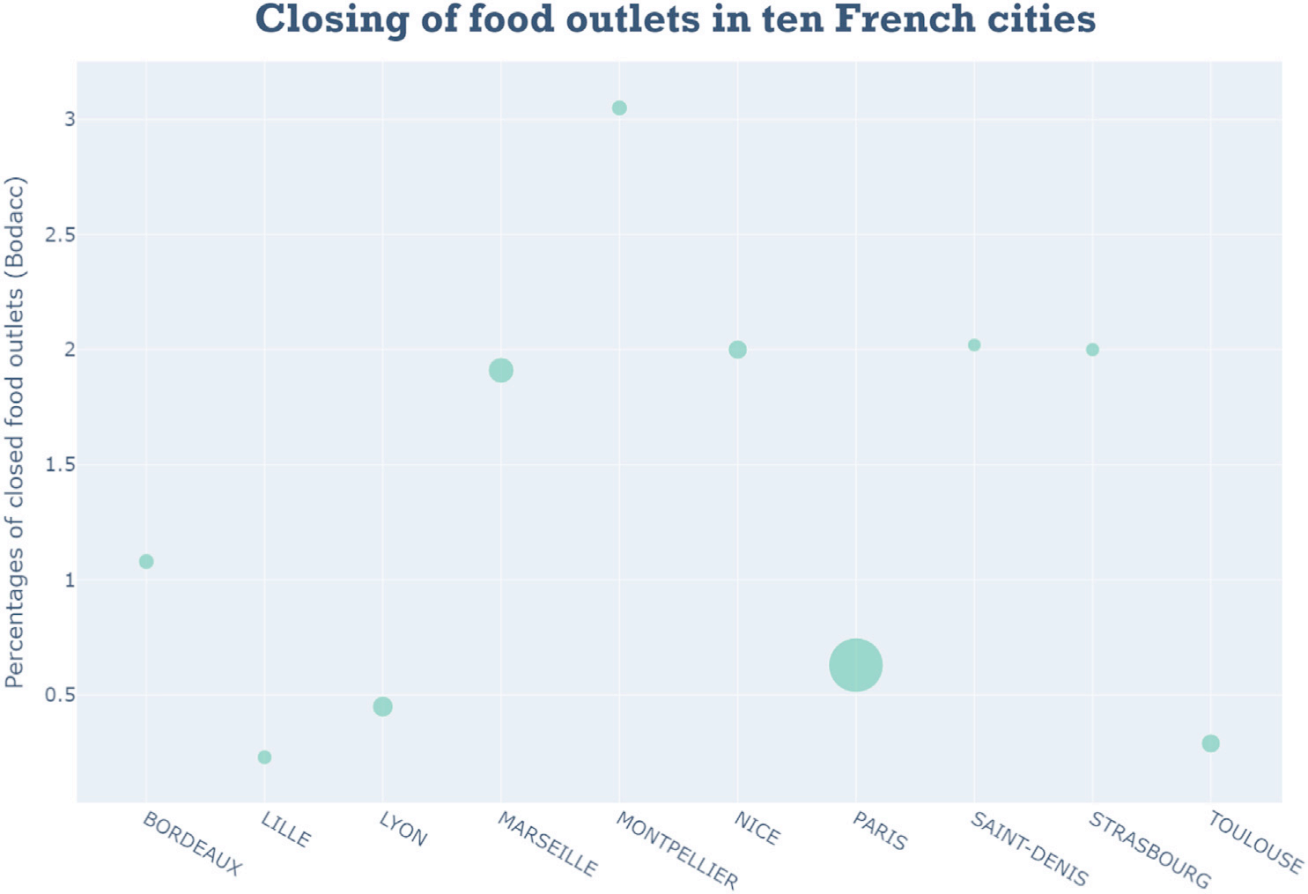
Dynamics of food outlets in 10 French towns

As Fig. 4 illustrates, Paris leads, with a significant number of food outlets (39,464), followed by Marseille (8,917) and Lyon (5,819), consistent with these being France’s three largest cities. While Paris has the highest number of closed food outlets (250), the percentage of closures remains relatively low (0.63 %). Similarly, Lyon (0.45 %), Toulouse (0.29 %), and Lille (0.23 %) exhibit low closure rates. In contrast, Montpellier (3.05 %), Saint-Denis (2.02 %), and Nice (2 %) have significantly higher percentages of closures.

Furthermore, initiating this database in September 2024 enables us to analyze the evolution of food outlets distribution over time according to socio-demographic and geographic changes. Those evolutions would be monthly based oriented to follow up the rhythm of the SIRENE 3.11 data base.

## Limitations

The database has many limitations. Firstly, the SIRENE 3.11 database, the basis for the construction of the work carried out, is due to be upgraded to version 4, where changes will be made to the variables. Even if they are minor, these changes will have to be taken into account and scrutinised so that the database can continue to be used and improved.

One of the intrinsic limitations of the databases used, especially in the case of SIRENE 3.11, is the fact that the geographical coordinates are not fully filled, although version 4 of Sirene is supposed to remedy this shortcoming.

## Ethics Statement

Authors have read and follow the ethical requirements for publication in Data in Brief and confirming that the current work does not involve human subjects, animal experiments, or any data collected from social media platforms.

## CRediT authorship contribution statement

**Quentin Creurer:** Conceptualization, Methodology, Software. **Simon Vonthron:** Methodology, Writing – review & editing. **Mohamed Hilal:** Methodology, Validation, Supervision. **Helene Charreire:** Writing – review & editing, Supervision. **Claude Napoleone:** Supervision. **Manon Pradere:** Visualization, Investigation. **Esther Sanz-Sanz:** Supervision, Funding acquisition.

## Data Availability

French-Food-Outlets-ScriptFrench-Food-Outlets-Script (Original data).

## Appendix Supplementary materials

Image, application 1
