# Supplementary material for: Decoding France’s food environment combining data bases to characterize the food environment in France
Source: Data Brief. 2025 Jul 11;62:111850. doi: 10.1016/j.dib.2025.111850 (PMC12396249; doi:10.1016/j.dib.2025.111850)
Supplement: Supplementary file 1 [file mmc1.pdf]

LICENCE OUVERTE

OPEN LICENCE

You may re-use the « Information » made available by the « Producer » under the freedoms and the conditions specified by this licence.

RE-USE OF INFORMATION UNDER THIS LICENCE

The « Producer » grants the « Re-user » a worldwide, perpetual, free of charge, non-exclusive, personal right to use the « Information » subject to this licence under the freedoms and the conditions set out below.

YOU ARE FREE TO RE-USE THE « INFORMATION » :

- To reproduce, copy, publish and transmit the « Information » ;
- To disseminate and redistribute the « Information » ;
- To adapt, modify, transform and extract from the « Information », for instance to build upon it in order to create « Derivative information » ;
- To exploit the « Information » commercially, for example, by combining it with other « Information », or by including it in your own product or application.

YOU MUST, WHERE YOU DO ANY OF THE ABOVE :

- Attribute the « Information » by acknowledging its source (at least the name of the « Producer ») and the date on which it was last updated.

  The « Re-user » may fulfil this condition by providing one or more hypertext links (URL) referring to the « Information » and effectively acknowledging its source.

  This attribution shall not suggest any official status or endorsement, by the « Producer » or any other public entity, of the « Re-user » or the re-use of the « Information ».

LIABILITY

The « Information » is made available as produced or received by the « Producer », without any other express or tacit guarantee not specified in this licence.

The « Producer » guarantees that it makes the « Information » available free of charge, under the freedoms and the conditions defined by this licence. It cannot guarantee that the « Information » does not contain any error or irregularity. It does not guarantee the continued supply of the « Information ». It shall not be liable for any loss, injury or damage of any kind caused to third parties resulting from its re-use.

The « Re-user » is solely responsible for its re-use of the « Information ». The re-use shall not mislead third parties or misrepresent the content of the « Information », its source and its time of last update.

INTELLECTUAL PROPERTY RIGHTS

The « Producer » guarantees that the « Information » is not subject to any « Intellectual property rights » belonging to third parties.

« Intellectual property rights » that might be held by the « Producer » over documents containing the « Information » do not impede the free re-use of the « Information ». Where the « Producer » holds « Intellectual property rights » over documents which contain the « Information », it assigns them, on a non-exclusive basis, free of charge, worldwide and for the entire term of the « Intellectual property rights », to the « Re-user », who may make use thereof in accordance with the freedoms and conditions defined in this licence.

COMPATIBILITY OF THE LICENCE

To facilitate the re-use of the « Information », this licence has been designed to be compatible with any licence which requires at least the attribution of the « Information ». For instance, it is compatible with the « Open Government Licence » (OGL) of the United Kingdom, the « Creative Commons Attribution 2.0 » (CC-BY 2.0) licence of Creative Commons and the « Open Data Commons Attribution » (ODC-BY) licence of the Open Knowledge Foundation.

APPLICABLE LAW

This licence is governed by French law.

DEFINITIONS

INTELLECTUAL PROPERTY RIGHTS*
« Intellectual property rights » means any rights identified as such by the French Intellectual Property Code (copyright, related rights, and sui generis rights over databases).

INFORMATION*
« Information » means data or information proposed for re-use with the freedoms and under the conditions of this licence.

DERIVATIVE INFORMATION*
« Derivative information » means new data or information which has been created either directly from the « Information », or using a combination of the « Information » and other data or information not subject to this licence.

PRODUCER*
« Producer » means the entity which produces the « Information » and opens it to re-use under the freedoms and the conditions of this licence.

RE-USER*
« Re-user » means any natural or legal person who re-uses the « Information » in accordance with the freedoms and the conditions of this licence.

ABOUT THE OPEN LICENCE

Etalab is the task force under the French Prime Minister's authority leading Open Government Data policy for France. Etalab introduces the Open Licence to facilitate and encourage easy reuse of public sector information – as defined by French Law – free of charge and as broadly as possible,

> *Within the scope of their public service missions, public sector bodies produce and receive public sector information, which may be re-used by any natural or legal person.*
>
> *Under the terms of French Law, are not considered public sector information : information the communication of which is not a right under information access legislation ; information contained in documents produced or received by public sector bodies exercising a public service of industrial or commercial character ; and information contained in documents over which third parties hold intellectual property rights,*
>
> *Information which contains personal data is not considered to be public sector information re-usable under the terms of French Law – except where persons on which data is collected have agreed to its reuse, where this data has been rendered anonymous by the public sector bodies, or where a legal or statutory provision permits its re-use (in these three cases, re-use is subject to compliance with French privacy protection legislation).*

This licence is version 1.0 of the Open Licence. Etalab may, from time to time, offer new versions of the Open Licence. However, re-users may continue to re-use information available under this licence if they wish to do so.

etalab gouv.fr
data.gouv.fr BETA innovation transparence ouverture
